# Antioxidant and Anti-Inflammatory Properties of Hydrolyzed Royal Jelly Peptide in Human Dermal Fibroblasts: Implications for Skin Health and Care Applications

**DOI:** 10.3390/bioengineering11050496

**Published:** 2024-05-16

**Authors:** Chang-Yu Yan, Qian-Qian Zhu, Cheng-Xi Guan, Gui-Lan Xiong, Xin-Xing Chen, Hai-Biao Gong, Jia-Wei Li, Shu-Hua Ouyang, Hiroshi Kurihara, Yi-Fang Li, Rong-Rong He

**Affiliations:** 1 Guangdong Engineering Research Center of Traditional Chinese Medicine & Disease Susceptibility/Guangdong-Hong Kong-Macao Universities Joint Laboratory for the Internationalization of TCM/Guangzhou Key Laboratory of Traditional Chinese Medicine & Disease Susceptibility/International Cooperative Laboratory of Traditional Chinese Medicine Modernization and Innovative Drug Development of Chinese Ministry of Education (MOE)/Guangdong Province Key Laboratory of Pharmacodynamic Constituents of TCM and New Drugs Research/Key Laboratory of Bioactive Molecules and Druggability Assessment, Jinan University, Guangzhou 510632, Chinahiroshi_kurihara@163.com (H.K.); 2 Lihe (Zhuhai Hengqin) Biopharmaceutical Technology Co., Ltd., Zhuhai 519031, China; 3 Lihe (Macao) Pharmaceutical Technology Co., Ltd., Macao 999078, China

**Keywords:** royal jelly peptide, antioxidant, anti-inflammatory, dermal fibroblasts, skin, oxidative stress, NLRP3 inflammasome

## Abstract

Hydrolyzed royal jelly peptide (RJP) has garnered attention for its health-promoting functions. However, the potential applications of RJP in skincare have not been fully explored. In this study, we prepared RJP through the enzymatic hydrolysis of royal jelly protein with trypsin and investigated its antioxidant and anti-inflammatory properties on primary human dermal fibroblasts (HDFs). Our results demonstrate that RJP effectively inhibits oxidative damage induced by H_2_O_2_ and lipid peroxidation triggered by AAPH and *t*-BuOOH in HDFs. This effect may be attributed to the ability of RJP to enhance the level of glutathione and the activities of catalase and glutathione peroxidase 4, as well as its excellent iron chelating capacity. Furthermore, RJP modulates the NLRP3 inflammasome-mediated inflammatory response in HDFs, suppressing the mRNA expressions of NLRP3 and IL-1β in the primer stage induced by LPS and the release of mature IL-1β induced by ATP, monosodium urate, or nigericin in the activation stage. RJP also represses the expressions of COX2 and iNOS induced by LPS. Finally, we reveal that RJP exhibits superior antioxidant and anti-inflammatory properties over unhydrolyzed royal jelly protein. These findings suggest that RJP exerts protective effects on skin cells through antioxidative and anti-inflammatory mechanisms, indicating its promise for potential therapeutic avenues for managing oxidative stress and inflammation-related skin disorders.

## 1. Introduction

The skin, the largest organ of the human body, serves multiple vital functions including protection against external threats, the regulation of body temperature, sensation facilitation, immune defense support, vitamin D synthesis, moisture retention, and waste product excretion [1]. Structurally, skin consists of three layers: the outer layer epidermis, the middle layer dermis, and the innermost layer hypodermis. The epidermis is primarily composed of keratinocytes, accounting for 95% of cells, with resident dendritic cells making up the remaining 5%, including melanocytes, Langerhans cells, and Merkel cells, forming the first-line-of-defense barrier [2]. Beneath the epidermis lies the dermis, a 1–2 mm thick layer rich in fibroblasts, which provides crucial support to the skin and its appendages. Fibroblasts maintain skin plasticity by the synthesis and secretion of various extracellular matrix (ECM) components, such as collagen and elastic fibers, which account for 70% of skin dry weight [3]. The dysfunction of dermal fibroblasts can lead to wrinkles and skin sagging as a consequence of a decreased production of the ECM, which drives skin aging [4]. Additionally, fibroblasts contribute to wound healing by depositing the ECM, facilitating tissue remodeling and repair [5]. Therefore, regulating the activity and functional homeostasis of dermal fibroblasts is an important strategy for improving the skin condition.

Dermal fibroblasts are susceptible to various oxidative and inflammatory stimuli, leading to cellular imbalance and damage [6,7]. Ultraviolet A (UVA), with a longer wavelength (320–400 nm) compared to UVB, can penetrate the epidermis and reach the dermal layer, causing the accumulation of reactive oxygen species (ROS) within fibroblasts [8,9]. In addition, certain endogenous factors can also lead to the accumulation of ROS, such as an increased mitochondrial leakage, elevated respiration, and higher O_2_ concentration [10]. While the antioxidant system, comprising enzymatic and non-enzymatic antioxidants in the dermis, can neutralize ROS, prolonged or excessive exposure will exceed the tolerance threshold, resulting in extensive cellular oxidative damage [11]. Protein and lipid oxidation can initiate the subsequent degradation or accumulation of toxic products, potentially leading to cell senescence or death [12,13]. Skin diseases associated with chronic inflammation, such as psoriasis and atopic dermatitis, can expedite the ECM breakdown by stimulating the production of matrix metalloproteinases (MMPs), enzymes that degrade collagen and elastin [14]. This is due to the transcriptional activation of MMPs in fibroblasts by inflammatory mediators like TNF-α, which can also induce premature senescence of fibroblasts, thereby inhibiting their function [15,16]. Furthermore, dermal fibroblasts themselves exhibit inflammatory response activity upon being exposed to external stimuli, leading to the secretion of pro-inflammatory cytokines and chemokines, which attract immune cells to the inflammation site, perpetuating the inflammatory reaction and causing further tissue damage [17,18].

Royal jelly is a secretion produced by the glands in the hypopharynx of nurse bees and is used as a diet for queen bees and larvae. Its primary constituents encompass water (60–70%), proteins (9–18%), carbohydrates (7–18%), and lipids (3–8%) [19]. Notably, the protein content in royal jelly serves as a significant nutritional component, with major royal jelly protein (MRJP) accounting for approximately 90%, and the identification of nine MRJPs (MRJP1-9) has been reported [20]. Recent studies have elucidated the diverse beneficial properties of royal jelly peptide (RJP) obtained through enzymatic hydrolysis, demonstrating its anti-hypertensive, antibacterial, antioxidative, and anti-aging activities [21]. However, research on the application of RJP in skincare remains limited. In this study, we investigated the antioxidant and anti-inflammatory properties of pancreatic trypsin hydrolyzed RJP in primary human dermal fibroblasts (HDFs) subjected to H_2_O_2_-, AAPH-, or *t*-BuOOH-induced oxidative stress and LPS-induced inflammation conditions. Our data indicate that RJP exhibits exceptional antioxidant and anti-inflammatory activities in HDFs, suggesting promising benefits for dermatological health and care.

## 2. Materials and Methods

### 2.1. Preparation of Hydrolyzed RIP

Freeze-dried royal jelly powder was obtained from Zhejiang Jiangshan Bee Enterprise Co., Ltd. (Quzhou, China). The royal jelly powder was dissolved in distilled water at a ratio of 1:5 (g:mL), and the pH was adjusted to 10.0 using NaOH. The solution was continuously stirred for 30 min, followed by centrifugation at 5000 rpm for 10 min to collect the supernatant. The pH was then adjusted to 3.5 with HCl to precipitate proteins, which were collected by centrifugation at 5000 rpm for 10 min. The precipitate was reconstituted in distilled water, and the pH was adjusted to 7.0. After centrifugation at 5000 rpm for 10 min, the royal jelly protein was collected.

The royal jelly protein was mixed with distilled water at a ratio of 1:20 (g: mL), and the pH was adjusted to 8.0. To this mixture, 5000 U/g of pancreatic trypsin (PTN 6.0 S, Novozymes (China) Biotechnology, Tianjin, China) was added, and the mixture was stirred well for 5 h at 50 °C. Finally, the hydrolysates were heat-treated at 95 °C for 10 min to inactivate the enzymes, followed by centrifugation at 10,000 rpm at 4 °C for 10 min. The resulting supernatant was freeze-dried to obtain RJP. The amino acid composition of RJP was determined by an automatic amino acid analyzer (S 7130, Sykam Company, Munich, Germany), and the result is presented in Table 1.

### 2.2. Isolation and Cultivation of Primary HDFs

HDFs were derived from foreskin tissues of adult males undergoing voluntary circumcision. Briefly, foreskin tissues were disinfected in 75% ethanol, washed with PBS, and subsequently, the subcutaneous fat and capillaries were removed. The remaining skin tissues were digested with 5 mg/mL of dispase II (04942078001, Roche, Mannheim, Germany) overnight at 4 °C to separate the epidermis and dermis. The dermis was sectioned into approximately 1 mm^2^ pieces and cultured in DMEM supplemented with 10% FBS and 1% penicillin–streptomycin. Tissue cultures were maintained with regular medium changes every 3 days and monitored daily under an optical microscope until a confluent monolayer was formed. HDFs (passage 1) were then collected. This study was conducted according to the guidelines of the Declaration of Helsinki and approved by the ethics committee of Jinan University (Approval ID: JNUKY-2023-0056).

### 2.3. Cell Viability

HDFs were seeded into 96-well plates and treated with H_2_O_2_ (300 μM) and RJP (5–200 μg/mL) for 12 h. An MTT solution (5 mg/mL) was added to each well, and the plates were further incubated at 37 °C for 3 h. The formazan crystals were solubilized with DMSO, and the absorbance was measured at 570 nm using a microplate reader (Synergy HT, BioTek, Winooski, VT, USA).

### 2.4. Detection of ROS and Lipid ROS

HDFs were seeded in 6-well plates at a density of 300,000 cells per well. Co-treatment with H_2_O_2_ (300 μM) and RJP (100 or 200 μg/mL) was carried out for 2 h. ROS staining was performed using a 10 μM H_2_DCFDA probe (S0033S, Beyotime Biotechnology, Shanghai, China) for 30 min, followed by fluorescence signal detection using fluorescence microscopy with the FITC channel (Olympus IX51, Olympus, Tokyo, Japan). For the assessment of intracellular lipid ROS, HDFs were co-treated with AAPH (300 μM) or *t*-BuOOH (100 μM) and RJP (100 or 200 μg/mL) for 2 h. Subsequently, cells were incubated with a 2 μM C11-BODIPY 581/591 probe (D3861, Invitroge, Carlsbad, CA, USA) for 30 min and harvested by centrifugation at 800 rpm for 5 min. The cell pellets were resuspended in PBS for the detection of the FITC fluorescence signal using a flow cytometer system (CytoFLEX S, Beckman Coulter, CA, USA).

### 2.5. Measurement of Malondialdehyde (MDA) and Glutathione (GSH)

After centrifugation at 4 °C for 10 min at 12,000 rpm, the supernatant of HDFs’ lysates was collected. The protein levels were quantified using the BCA method. MDA and GSH levels were measured separately using commercial kits (S0131 and S0053, Beyotime Biotechnology) following the manufacturer’s instructions. Finally, the concentration of MDA was normalized to the protein content, and the ratio of reduced GSH to oxidized GSSG was calculated.

### 2.6. Evaluation of Glutathione Peroxidase 4 (GPX4) and Catalase (CAT) Activity

HDFs’ lysates were prepared using a non-denaturing lysis buffer (P0013, Beyotime Biotechnology), followed by centrifugation at 12,000 rpm for 10 min. The resulting supernatant was collected for assessing GPX4 and CAT activity using commercial assay kits (S0056 and S0051, Beyotime Biotechnology), according to the manufacturer’s instructions. The activity of GPX4 and CAT was then normalized based on the protein concentration.

### 2.7. Oxygen Radical Absorbance Capacity (ORAC) Assay

The ORAC assay was conducted following a previously described method [22]. Proteins in HDFs’ lysate were removed with 3% perchloric acid. HDFs’ sample (20 μL) or Trolox (20 μL) were added to a 96-well plate along with a phosphate buffer (20 μL) and fluorescence sodium (20 μL). Subsequently, 140 μL of AAPH was added to each well to initiate the reaction. The fluorescence intensity was measured every 4 min at 37 °C under a microplate reader (Synergy HT, BioTek), with excitation at 485 nm and emission at 527 nm. The ORAC value was calculated as a Trolox equivalent (eq.).

### 2.8. Iron Chelating Assay

The iron chelating ability of RJP was assessed using the Prussian blue assay. Briefly, 50 μL of the RJP solution was mixed with 50 μL of ferrous chloride (10 mM) and allowed to incubate for 10 min at room temperature. Subsequently, 50 μL of potassium ferricyanide (5 mM) was added to the mixture. The absorbance was measured at 680 nm using a microplate reader (Synergy HT, BioTek).

### 2.9. Measurement of Free Iron in HDFs

The supernatants obtained from HDFs’ lysates were incubated with a 2 μM FerroOrange probe (F374, Dojindo Laboratories, Kumamoto, Japan) at 37 °C for 30 min. The fluorescence intensity was then measured using a microplate reader (Synergy HT, BioTek) at an excitation/emission wavelength of 543/580 nm.

### 2.10. IL-1β Measurement by ELISA

Supernatants from HDFs’ cultures were harvested, and the levels of IL-1β were assessed using a Human IL-1 beta/IL-1F2 DuoSet ELISA Kit (DY201-05, R&D Systems, Minneapolis, MN, USA) following the manufacturer’s protocols.

### 2.11. Western Blot

HFDs were lysed using a RIPA lysis buffer (P0013B, Beyotime Biotechnology) supplemented with protease inhibitor cocktails (P1005, Beyotime Biotechnology). The protein content in the supernatant was detected using a BCA protein assay kit (23225, Thermo Scientific, Waltham, MA, USA). The proteins were then separated by SDS-PAGE and transferred onto PVDF membranes (IPVH00010, Merck Millipore, Burlington, MA, USA). Blots were probed overnight at 4 °C with a 4-hydroxynonenal (4-HNE) antibody (ab46545, Abcam, Cambridge, UK) or a GAPDH antibody (FD0063, Fude Biological Tech., Hangzhou, China), followed by incubation with HRP-labeled secondary antibodies (Rabbit, FDR007; Mouse, FDM007, Fude Biological Tech.) for 2 h at room temperature. The blots were detected using the FDbio-Dura ECL substrate (FD8020, Fude Biological Tech.) and visualized using an imaging system (Tanon-5200, Tianneng Life Science, Shanghai, China).

### 2.12. qPCR

Total RNA was extracted from HDFs with an RNAiso Plus regent (9109, TaKaRa, Otsu, Japan), followed by reverse transcription to cDNA using *Evo M-MLV* RT Premix (AG11706, Accurate Biotechnology, Changsha, China). A quantitative PCR analysis was conducted using a SYBR Green Premix *Pro tag* HS qPCR Kit (AG11701, Accurate Biotechnology) on a real-time PCR system (CFX Connect, Bio-Rad, CA, USA). GAPDH served as the internal control for normalizing the levels of target genes. The relative quantification of gene expression was calculated using the 2^−ΔΔCT^ method. Primer sequences are shown in Table 2.

### 2.13. Statistical Analysis

Statistical analyses were conducted using GraphPad Prism software (version 8.0.2). Data are expressed as means ± standard deviation (SD). Statistical differences were analyzed by one-way ANOVA followed by Dunnett’s multiple comparison test. *p* < 0.05 was considered statistically significant.

## 3. Results

### 3.1. RJP Inhibits Oxidative Damage Induced by H_2_O_2_

**Figure 1:**
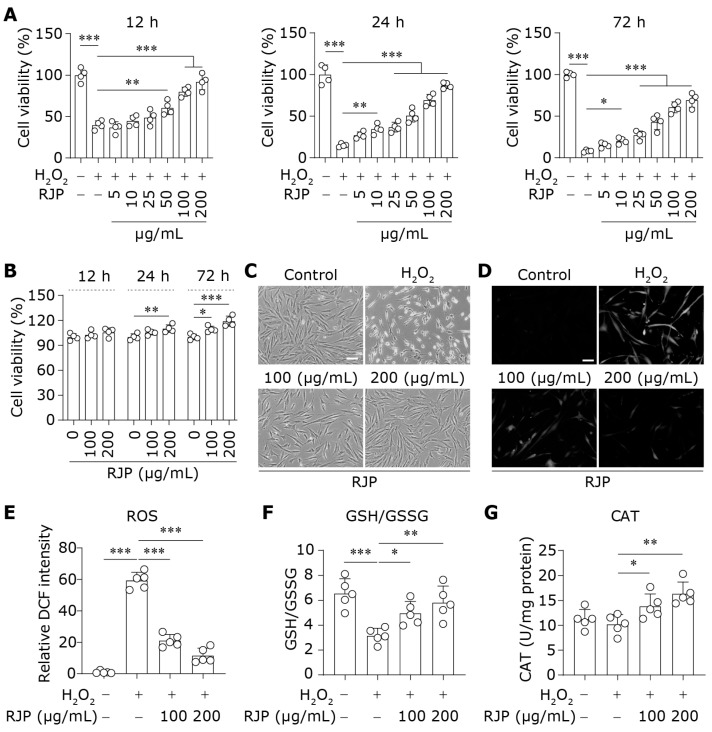
RJP inhibits oxidative damage induced by H_2_O_2_. (**A**) The cell viability of H_2_O_2_ (300 μM)-stressed HDFs for 12–72 h treated with RJP (5–200 μg/mL). *n* = 4 independent biological repeats. (**B**) The cell viability of normal HDFs treated with RJP (100 or 200 μg/mL) for 12–72 h. *n* = 4 independent biological repeats. (**C**) The morphological change in H_2_O_2_ (300 μM)-stressed HDFs for 12 h treated with RJP (100 or 200 μg/mL). The scale bar represents 100 μm. (**D**,**E**) The ROS level was detected by H_2_DCFDA staining and the fluorescent microscope in H_2_O_2_ (300 μM)-stressed HDFs for 2 h treated with RJP (100 or 200 μg/mL). The scale bar represents 100 μm. *n* = 5 independent biological repeats. (**F**,**G**) The ratio of GSH to GSSG and the activity of CAT in H_2_O_2_ (300 μM)-stressed HDFs for 6 h treated with RJP (100 or 200 μg/mL). *n* = 5 independent biological repeats. Data are presented as means ± SD. The statistical differences were analyzed by one-way ANOVA followed by Dunnett’s multiple comparison test with * *p* < 0.05, ** *p* < 0.01, and *** *p* < 0.001.

An oxidative damage model was established in HDFs using H_2_O_2_, and changes in cell viability were assessed following treatment with varying concentrations of RJP. As shown in Figure 1A, the cell viability increased with escalating doses of RJP after stimulation with H_2_O_2_ for 12 h, 24 h, and 72 h, indicating its potential in restoring cellular activity in H_2_O_2_-stressed HDFs. In addition, we evaluated the effect of RJP (100 and 200 μg/mL) on normal HDFs and found that RJP exhibited a very weak proliferative effect (Figure 1B). Microscopic observation revealed pronounced morphological alterations in HDFs exposed to H_2_O_2_, exhibiting a shriveled appearance compared to the control group. However, treatment with RJP restored cell morphology, with the RJP (200 μg/mL) group displaying negligible changes comparable to normal HDFs (Figure 1C). Subsequently, the H_2_DCFDA fluorescence probe was used to qualitatively assess intracellular ROS levels. As depicted in Figure 1D,E, ROS levels were markedly elevated in H_2_O_2_-exposed HDFs compared to control cells, which was attenuated with RJP treatment, suggesting the protective effect of RJP against oxidative-stress-induced damage. GSH functions by scavenging ROS, leading to its oxidation to glutathione disulfide (GSSG). The GSH/GSSG radio is an important indicator of oxidative stress [23]. H_2_O_2_ consumption depleted intracellular GSH, resulting in a decrease in the GSH/GSSG ratio. However, compared to the H_2_O_2_ group, RJP treatment prevented the depletion of GSH (Figure 1F). CAT is an enzyme responsible for the decomposition of H_2_O_2_ into water and oxygen to prevent its accumulation. Though CAT activity remained unchanged under H_2_O_2_ exposure, RJP treatment increased CAT activity, consequently accelerating the removal of H_2_O_2_ (Figure 1G). Taken together, these results suggest that RJP exhibits potent antioxidant effects and inhibits oxidative damage induced by H_2_O_2_ in HDFs.

### 3.2. RJP Counteracts Lipid Peroxidation Triggered by AAPH and t-BuOOH

**Figure 2:**
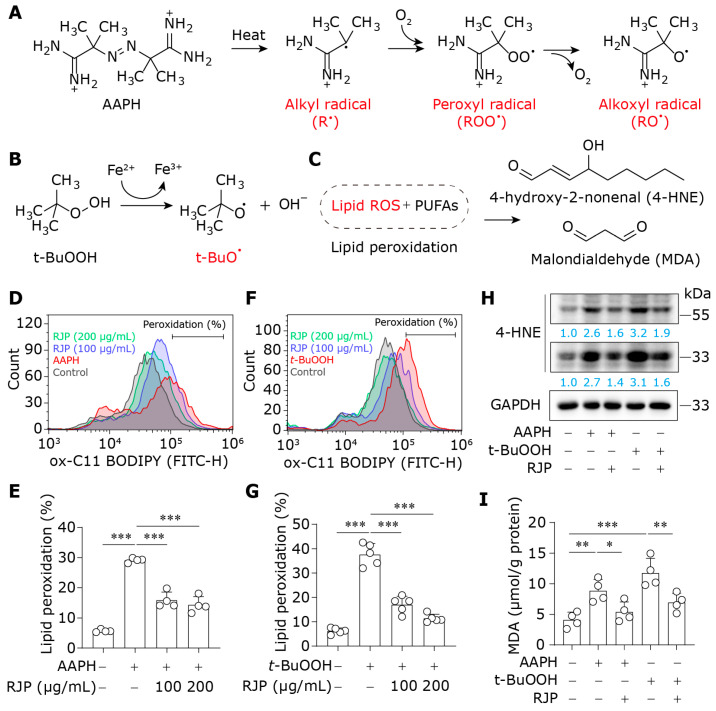
RJP counteracts lipid peroxidation triggered by AAPH and *t*-BuOOH. (**A**,**B**) The lipid ROS metabolism initiated by AAPH and *t*-BuOOH. (**C**) Lipid peroxidation leads to the generation of reactive aldehydes, including 4-HNE and MDA. (**D**,**E**) The lipid ROS was measured by C11-BODIPY staining and flow cytometry in AAPH (300 μM)-stressed HDFs for 2 h treated with RJP (100 or 200 μg/mL). *n* = 4 independent biological repeats. (**F**,**G**) The lipid ROS was measured by C11-BODIPY staining and flow cytometry in *t*-BuOOH (100 μM)-stressed HDFs for 2 h treated with RJP (100 or 200 μg/mL). *n* = 5 independent biological repeats. (**H**) The levels of 4-HNE in AAPH (300 μM)- or *t*-BuOOH (100 μM)-stressed HDFs for 6 h treated with RJP (200 μg/mL). The numbers under the bands indicate protein expression compared to the first lane on the left with GAPDH as the internal reference. (**I**) The levels of MDA in AAPH (300 μM)- or *t*-BuOOH (100 μM)-stressed HDFs for 6 h treated with RJP (200 μg/mL). *n* = 4 independent biological repeats. Data are presented as means ± SD. The statistical differences were analyzed by one-way ANOVA followed by Dunnett’s multiple comparison test with * *p* < 0.05, ** *p* < 0.01, and *** *p* < 0.001.

Next, we employed AAPH and *t*-BuOOH to induce lipid peroxidation in HDFs. The process by which they generate lipid ROS is illustrated in Figure 2A,B. These lipid ROS attack the polyunsaturated fatty acids (PUFAs) within cell membrane phospholipids, initiating a chain reaction of lipid peroxidation that yields a substantial amount of lipid peroxides, ultimately metabolizing into reactive aldehydes such as 4-HNE and MDA [24] (Figure 2C). The effects of RJP on lipid peroxidation in AAPH- or *t*-BuOOH-stressed HDFs were detected using flow cytometry and BODIPY 581/591 C11 staining, a fluorescent probe labeling lipid peroxide [25]. As shown in Figure 2D–G, lipid peroxidation levels in HDFs significantly escalated following AAPH or *t*-BuOOH stress, while treatment with RJP markedly attenuated the lipid peroxidation level. 4-HNE possesses electrophilic properties and can react with various proteins to form 4-HNE-protein adducts, which can be detected via a Western blot analysis. Following AAPH or *t*-BuOOH stress, the level of 4-HNE-protein adducts was upregulated. Nonetheless, compared with the model group, the abundance of 4-HNE-protein adducts decreased (Figure 2H). Subsequently, the level of MDA was detected, revealing similar results, underscoring the inhibitory effect of RJP on AAPH- or *t*-BuOOH-induced MDA elevation (Figure 2I). Overall, RJP counteracts lipid peroxidation triggered by AAPH and *t*-BuOOH in HDFs.

### 3.3. RJP Restores Antioxidative Capacity and Intracellular Iron Homeostasis in HDFs

**Figure 3:**
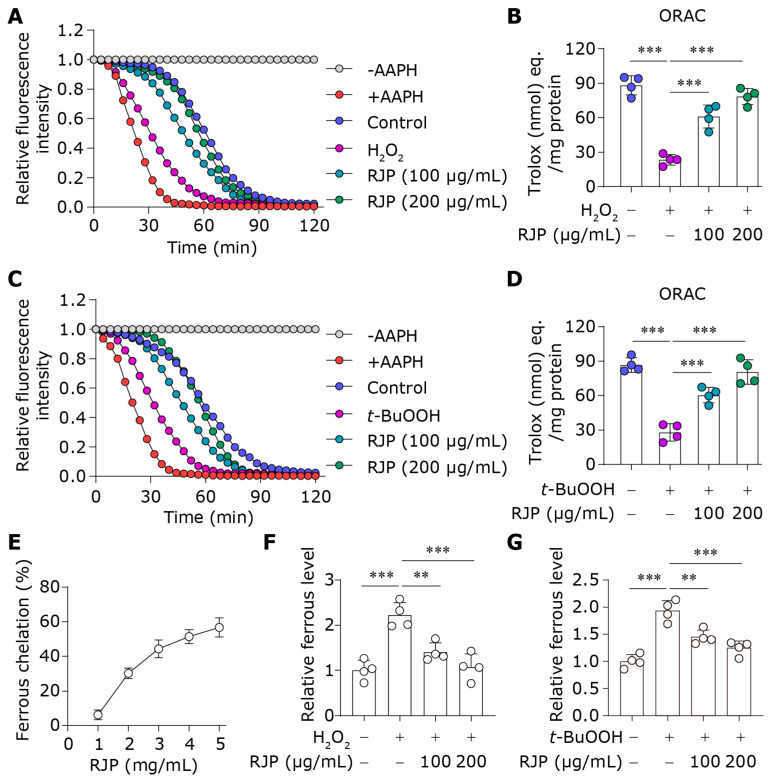
RJP restores antioxidative capacity and intracellular iron homeostasis in HDFs. (**A**,**B**) The fluorescence decay curve of uranine in the ORAC experiment, with the antioxidant values of HDFs’ lysate expressed as Trolox equivalents (eq.). HDFs were stressed with H_2_O_2_ (300 μM) for 6 h and treated with RJP (100 or 200 μg/mL). *n* = 4 independent biological repeats. (**C**,**D**) The fluorescence decay curve of uranine in the ORAC experiment, with the antioxidant values of HDFs’ lysate expressed as Trolox equivalents (eq.). HDFs were stressed with *t*-BuOOH (100 μM) for 6 h and treated with RJP (100 or 200 μg/mL). *n* = 4 independent biological repeats. (**E**) The iron chelating activity of RJP in a cell-free system. *n* = 4 independent biological repeats. (**F**,**G**) The levels of iron in H_2_O_2_ (300 μM)- or *t*-BuOOH (100 μM)-stressed HDFs for 6 h treated with RJP (100 or 200 μg/mL) were assessed using the FerroOrange probe. *n* = 4 independent biological repeats. Data are presented as means ± SD. The statistical differences were analyzed by one-way ANOVA followed by Dunnett’s multiple comparison test with ** *p* < 0.01 and *** *p* < 0.001.

To investigate the impact of RJP on the antioxidative capacity of HDFs, we evaluated the ability of HDFs’ lysates to scavenge free radicals by ORAC experiments. As depicted in Figure 3A,B, exposure to H_2_O_2_ significantly diminished the antioxidative activity of cell lysates. However, treatment with RJP progressively reinstated cellular capacity to counteract free radicals. A similar trend was observed in HDFs subjected to *t*-BuOOH stress (Figure 3C,D). In addition, our results revealed the excellent iron chelating activity of RJP in a cell-free system using the Prussian blue assay (Figure 3E). Moreover, a substantial elevation in intracellular free iron was observed in HDFs exposed to H_2_O_2_ and *t*-BuOOH. A previous study indicates that the exogenous H_2_O_2_ stimulation increases intracellular free iron through the regulation of iron metabolism proteins such as inhibiting the levels of Ferritin and Ferroportin to reduce iron storage and export [26]. Notably, treatment with RJP markedly reduced the levels of free iron, indicating its effective iron chelation activity within the cells (Figure 3F,G). The above results reveal the capacity of RJP to restore the antioxidative capacity and modulate intracellular iron homeostasis in HDFs under oxidative stress.

### 3.4. RJP Augments the Activity of the Lipid Peroxidation Inhibitor GPX4

**Figure 4:**
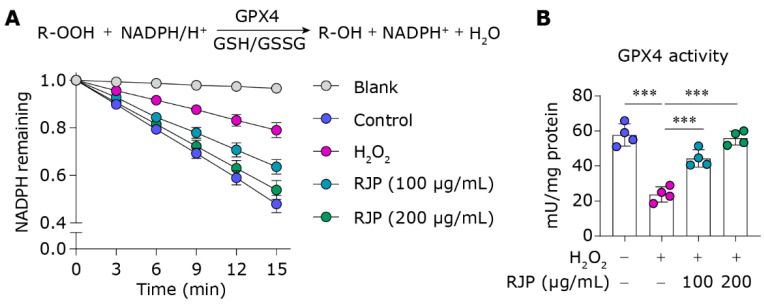
RJP augments the activity of the lipid peroxidation inhibitor GPX4. (**A**) The decay curve of NADPH levels, normalized to the baseline at time point 0 min. (**B**) The GPX4 activity of H_2_O_2_ (300 μM)-stressed HDFs for 6 h treated with RJP (100 or 200 μg/mL). *n* = 4 independent biological repeats. Data are presented as means ± SD. The statistical differences were analyzed by one-way ANOVA followed by Dunnett’s multiple comparison test with *** *p* < 0.001.

GPX4, an antioxidant enzyme, plays a pivotal role in mitigating lipid peroxidation by catalyzing the reduction of toxic lipid hydroperoxides to non-toxic alcohols [27]. In our study, we evaluated the impact of RJP on GPX4 enzyme activity. During the reduction reaction, GPX4 requires the consumption of NADPH. Therefore, changes in NADPH levels in the reaction system serve as a representation of its enzymatic activity. Under conditions of oxidative stress induced by H_2_O_2_, the activity of GPX4 was significantly compromised (Figure 4A,B). This is likely due to the depletion in GSH, an essential cofactor for GPX4 function (Figure 1F). However, treatment with RJP restored the activity of GPX4. This suggests that RJP has the ability to enhance GPX4 enzyme activity, which contributes to its protective effects against lipid peroxidation-induced damage.

### 3.5. RJP Suppresses the Expression of NLRP3 Inflammasome Components Initiated by LPS

**Figure 5:**
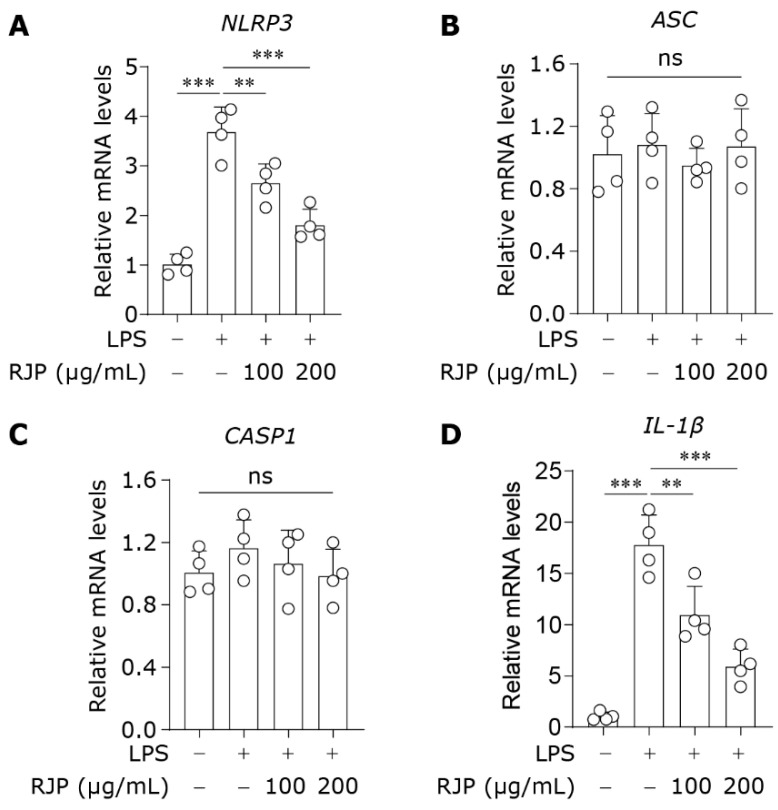
RJP suppresses the expression of NLRP3 inflammasome components initiated by LPS. HDFs were stimulated with LPS (10 μg/mL) for 12 h and co-treated with RJP (100 or 200 μg/mL). The gene expressions of NLRP3 inflammasome components, including (**A**) *NLRP3*, (**B**) *ASC*, (**C**) *CASP1*, and (**D**) *IL-1β*, were detected by qPCR with special primers. *n* = 4 independent biological repeats. Data are presented as means ± SD. The statistical differences were analyzed by one-way ANOVA followed by Dunnett’s multiple comparison test with ** *p* < 0.01 and *** *p* < 0.001; ns indicates not significant.

Previous research has demonstrated that HDFs express components of NLRP3 inflammasomes and exhibit inflammatory responses upon exposure to advanced glycation end products [28]. Our objective is to investigate the anti-inflammatory properties of RJP in HDFs by targeting the NLRP3 inflammasome. The NLRP3 inflammasome is a multiprotein complex composed of the pattern recognition receptor protein NLRP3, the adapter protein ASC, and the effector protein caspase-1 [29]. The activation of the NLRP3 inflammasome involves two primary stages: the priming stage, characterized by the upregulation of NLRP3 and other inflammasome components in response to various stimuli such as microbial products or cellular damage signals, and subsequent assembly of the NLRP3–ASC–caspase-1 complex upon secondary stimulation to activate pro-caspase-1 into cleaved caspase-1 [30]. LPS is a classic priming signal through binding to TLR4. Thus, we evaluated the effects of RJP on the expression of NLRP3 inflammasome components in an LPS-induced model by qPCR. As illustrated in Figure 5A–C, LPS significantly increased the mRNA expression of the *NLRP3* but did not affect the mRNA levels of *ASC* and *CASP1* (*caspase-1*). However, compared to the LPS group, RJP significantly inhibited the expression of *NLRP3* without affecting the other two components. The activation of caspase-1 leads to the cleavage of pro-IL-1β into its active form, mature IL-1β, which is then released into the extracellular space [31]. Therefore, an adequate expression of pro-IL-1β at the priming stage also determines the release of IL-1β mediated by the NLRP3 inflammasome. Correspondingly, RJP markedly suppressed the mRNA expression of *IL-1β* in LPS-induced HDFs (Figure 5D). Altogether, these findings indicate that RJP suppresses the expression of NLRP3 inflammasome components initiated by LPS.

### 3.6. RJP Hinders the Activation of NLRP3 Inflammasome

**Figure 6:**
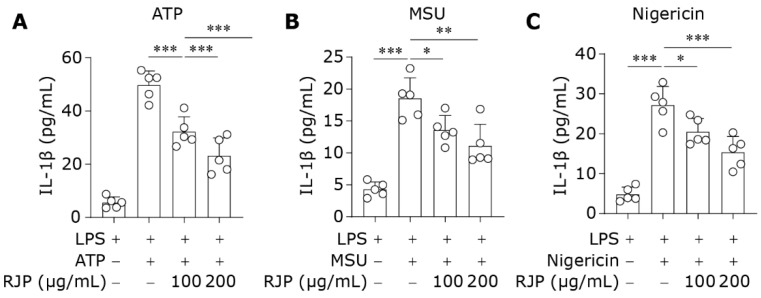
RJP hinders the activation of the NLRP3 inflammasome. HDFs were stimulated with LPS (10 μg/mL) for 12 h and then co-treated with RJP (100 or 200 μg/mL) for another 3 h. Next, (**A**) ATP (5 mM, 2 h), (**B**) MSU (0.5 mg/mL, 6 h), or (**C**) nigericin (10 μM, 2 h) was used to induce the activation of the NLRP3 inflammasome. The culture supernatant was collected for the determination of IL-1β by ELISA. *n* = 5 independent biological repeats. Data are presented as means ± SD. The statistical differences were analyzed by one-way ANOVA followed by Dunnett’s multiple comparison test with * *p* < 0.05, ** *p* < 0.01, and *** *p* < 0.001.

The NLRP3 inflammasome can be activated by various structurally and functionally distinct secondary signals, including ATP, monosodium urate (MSU), and nigericin, which leads to the maturation and release of IL-1β [32,33]. To assess the inhibitory effect of RJP on NLRP3 inflammasome activation, HDFs were first stimulated with LPS (10 μg/mL) for 12 h, followed by treatment with ATP (5 mM, 2 h), MSU (0.5 mg/mL, 6 h), or nigericin (10 μM, 2 h) to induce the release of IL-1β. The levels of IL-1β in the culture supernatant were measured by ELISA. As shown in Figure 6A–C, ATP, MSU, and nigericin led to the release of IL-1β in LPS-primed HDFs; however, treatment with RJP after LPS stimulation reduced the levels of IL-1β, indicating its direct suppressive effect on the second stage of NLRP3 inflammasome activation. These results suggest that RJP effectively hinders the activation of the NLRP3 inflammasome in HDFs.

### 3.7. RJP Represses COX2 and iNOS Expressions in LPS-Stimulated HDFs

**Figure 7:**
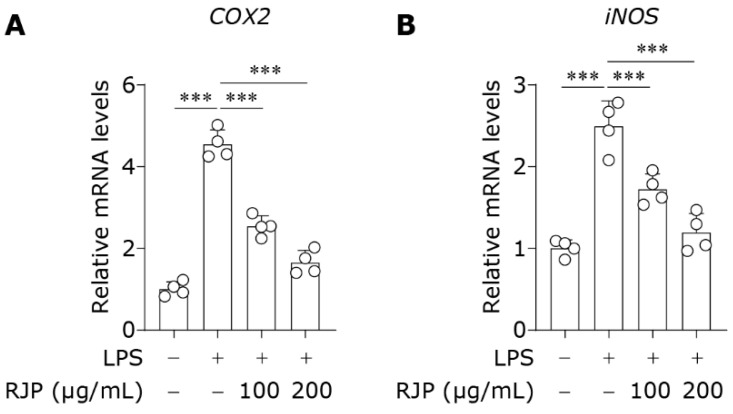
RJP represses COX2 and iNOS expressions in LPS-stimulated HDFs. HDFs were stimulated with LPS (10 μg/mL) for 12 h and co-treated with RJP (100 or 200 μg/mL). The gene expressions of (**A**) *COX2* and (**B**) *iNOS* were detected by qPCR. *n* = 4 independent biological repeats. Data are presented as means ± SD. The statistical differences were analyzed by one-way ANOVA followed by Dunnett’s multiple comparison test with *** *p* < 0.001.

LPS induces the expression of cyclooxygenase-2 (COX-2) and inducible nitric oxide synthase (iNOS), two crucial inflammatory enzymes responsible for the conversion of arachidonic acid to prostaglandins and synthesis of nitric oxide (NO) [34]. Excessive prostaglandins and NO can exacerbate inflammatory processes, leading to vasodilation, increased vascular permeability, and recruitment of immune cells, and the induction of cytokines, thereby delaying the reparative process or worsening skin damage [35,36]. When exposed to LPS, HDFs upregulated *COX-2* and *iNOS* expression, while treatment with RJP significantly decreased their mRNA levels (Figure 7A,B). The findings indicate that RJP modulates COX2 and iNOS expressions in LPS-stimulated HDFs.

### 3.8. RJP Exhibits Superior Antioxidant and Anti-Inflammatory Properties over Royal Jelly Protein

**Figure 8:**
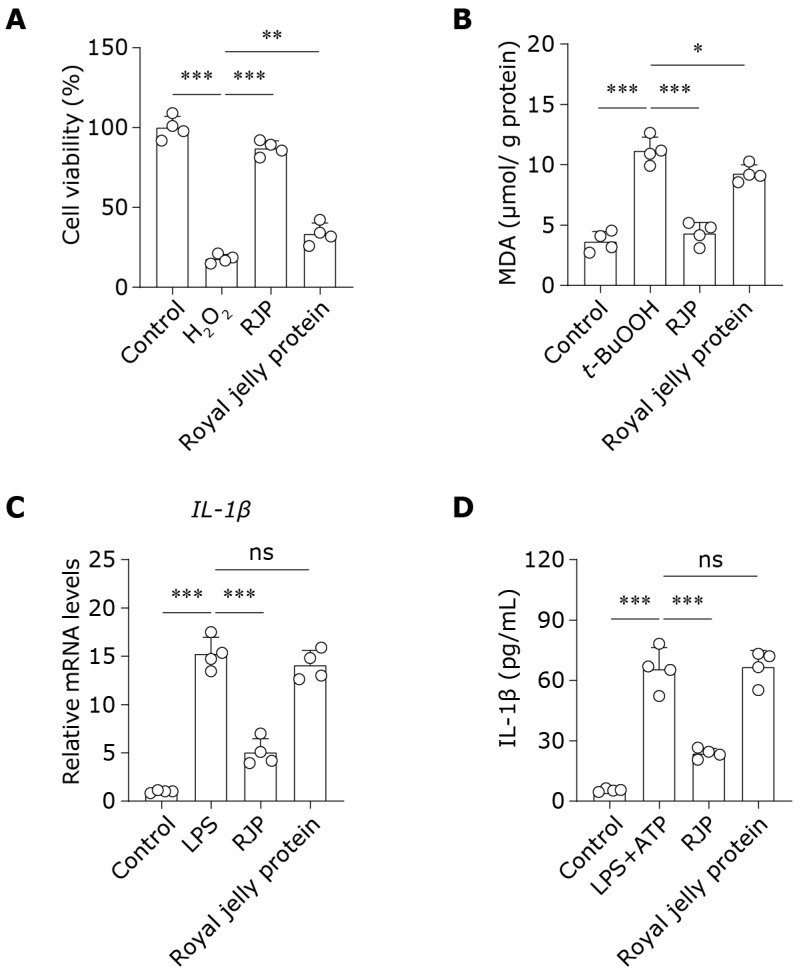
RJP exhibits superior antioxidant and anti-inflammatory properties over royal jelly protein. (**A**) The cell viability of H_2_O_2_ (300 μM)-stressed HDFs for 24 h treated with RJP (200 μg/mL) or royal jelly protein (200 μg/mL). *n* = 4 independent biological repeats. (**B**) The levels of MDA in *t*-BuOOH (100 μM)-stressed HDFs for 6 h treated with RJP (200 μg/mL) or royal jelly protein (200 μg/mL). *n* = 4 independent biological repeats. (**C**) The gene expressions of *IL-1β* were detected by qPCR in HDFs stimulated with LPS (10 μg/mL) for 12 h and co-treated with RJP (200 μg/mL) or royal jelly protein (200 μg/mL). *n* = 4 independent biological repeats. (**D**) HDFs were stimulated with LPS (10 μg/mL) for 12 h and then co-treated with RJP (200 μg/mL) or royal jelly protein (200 μg/mL) for another 3 h. Next, ATP (5 mM, 2 h) was used to induce the activation of the NLRP3 inflammasome. The culture supernatant was collected for the determination of IL-1β by ELISA. *n* = 4 independent biological repeats. Data are presented as means ± SD. The statistical differences were analyzed by one-way ANOVA followed by Dunnett’s multiple comparison test with * *p* < 0.05, ** *p* < 0.01, and *** *p* < 0.001; ns indicates not significant.

Finally, we compared the antioxidant and anti-inflammatory activities of RJP and unhydrolyzed royal jelly protein in HDFs. As shown in Figure 8A,B, compared to RJP, intact royal jelly protein exhibited only a very mild inhibitory effect on H_2_O_2_-induced cell death and *t*-BuOOH-induced lipid peroxidation product MDA accumulation. Additionally, similar results were obtained in the anti-inflammatory experiments. Unhydrolyzed royal jelly protein barely altered the elevation in *IL-1β* gene expression induced by LPS and the extracellular release of IL-1β induced by LPS + ATP (Figure 8C,D). These results collectively demonstrate that RJP exhibits superior antioxidant and anti-inflammatory properties over royal jelly protein.

## 4. Discussion

Royal jelly, in addition to being a valuable nutritional substance, has garnered widespread attention and research interest for its health-promoting properties. Previous studies have indicated its role in maintaining skin homeostasis. The topical application of cream containing 10% royal jelly effectively reduced levels of the inflammatory mediator TNF-α in UVB-induced rat skin tissue [37]. In a rat model of skin aging induced by estrogen deficiency, royal jelly demonstrated an ability to enhance collagen production in skin tissue [38]. Furthermore, royal jelly protected human skin fibroblasts against UVB-induced photoaging by enhancing collagen synthesis, with the active ingredient believed to be 10-hydroxy-2-decenoic acid (10-HDA) [39]. In fact, the absorption of high-molecular-weight proteins by cells presents a significant challenge, as proteins in royal jelly struggle to penetrate the cell membrane to exert their effects. Enzymatic hydrolysis can reduce the molecular weight of royal jelly proteins and improve the bioactivity [40]. Thus, in this study, we utilized pancreatic trypsin hydrolysis to prepare low-molecular-weight RJP, and revealed its excellent antioxidant and anti-inflammatory properties in HDFs. Our findings provide insight into the antioxidative and anti-inflammatory properties of RJP in skin cells, highlighting its potential applications in dermatological health and care.

H_2_O_2_ is a type of ROS inducer, which triggers oxidative stress and cell death in mouse and human skin fibroblasts [41,42,43]. Our investigation revealed that RJP exhibited remarkable antioxidative effects, protecting HDFs from oxidative damage induced by H_2_O_2_ accompanied by an enhanced activity of CAT and elevated levels of GSH. Indeed, the glutathione-dependent pathways represent another crucial mechanism for cellular H_2_O_2_ consumption, involving enzymes such as glutathione peroxidase and glutathione reductase [44]. Lipid peroxidation is a significant mechanism of cell damage induced by H_2_O_2_, where uncontrolled lipid peroxidation ultimately leads to irreversible damage to cell membranes, thus triggering ferroptotic cell death [45]. We found that RJP significantly inhibited lipid ROS induced by AAPH and *t*-BuOOH, as well as the levels of lipid peroxidation metabolites MDA and 4-HNE. Several studies have demonstrated that AAPH, *t*-BuOOH, and 4-HNE can induce oxidative cell death [46,47,48]. Through ORAC experiments, RJP was found to enhance the clearance ability of lipid radicals of lysates of HDFs stressed by H_2_O_2_ and *t*-BuOOH. In cell-free studies, RJP exhibited potential for free radical scavenging and the inhibition of lipid peroxidation [49,50], which may be related to the presence of certain antioxidant amino acid residues in RJP. However, this alone does not imply that its ability can fully protect against cellular damage. In addition to the regulatory effects on the oxidative–reductive metabolism enzymes mentioned above, RJP also exhibits certain iron chelating activity to reduce the accessibility of free iron in the cells, which collectively contribute to reducing oxidative stress. Iron can catalyze the generation of hydroxyl radicals (HO**·**) with high redox potential through the Fenton reaction. HO**·** catalyzes lipid peroxidation by attacking PUFAs in a non-enzymatic manner. Therefore, chelating iron represents a crucial antioxidative strategy [51]. The excellent iron chelating activity of RJP effectively reduced the levels of free iron in cells, restoring iron homeostasis and helping to reduce the production of lipid peroxides.

Most skin disorders are accompanied by a chronic inflammatory response, believed to accelerate skin aging [52]. Our study unveiled the robust anti-inflammatory properties of RJP, demonstrated by its ability to suppress LPS-induced inflammation in HDFs. During the initiation phase of the NLRP3 inflammasome induced by LPS, RJP significantly inhibited the gene expressions of *NLRP3* and *IL-1β*. Considering that both are downstream target genes of NF-*κ*B, we speculate that RJP may exert its anti-inflammatory effects by inhibiting NF-*κ*B activation through the LPS-TLR4 signaling [53]. In other inflammatory models, royal jelly has been found to reduce tissue inflammation damage by inhibiting NF-*κ*B. You et al. observed that royal jelly attenuates LPS-induced inflammation in BV-2 microglial cells by modulating NF-*κ*B signaling [54]. Moreover, RJP inhibited two other inflammatory-related target genes of NF-*κ*B, *COX2* and *iNOS*, in LPS-stimulated HDFs. Furthermore, we found that RJP also modulates the activation phase of the NLRP3 inflammasome, suppressing the release of mature IL-1β, a crucial inflammatory mediator in skin diseases [55], induced by activating signals such as ATP, MSU, and nigericin. This stage involves multiple mechanisms, including the polymerization of NRLP3 and ASC, as well as post-translational modifications of NLRP3 protein [56]. Our previous study indicated that the natural product celastrol inhibits the K63-deubiquitination of NLRP3, exerting potent anti-inflammatory effects [57]. Additionally, the activation of NLRP3 inflammasomes also induces an inflammatory form of cell death known as pyroptosis, suggesting the potential protective effect of RJP against inflammatory damage in HDFs [58]. The anti-inflammatory action underscores the promising application of RJP as a therapeutic agent for addressing inflammatory skin conditions.

Our findings reveal that intact royal jelly protein exhibited only a minimal inhibitory effect on H_2_O_2_-induced cell death and *t*-BuOOH-induced lipid peroxidation. Additionally, unhydrolyzed royal jelly protein scarcely impacted the activation of the NLRP3 inflammasome. This suggests that enzymatic hydrolysis significantly enhances the activities of royal jelly protein, as previous studies indicate that enzymatic hydrolysis, resulting in the generation of small-molecular-weight peptides, not only confers significant advantages in cellular uptake but also releases specific amino acid sequences to exert activity [59,60]. Indeed, royal jelly protein is a widely accepted protein resource, and it is essential to enhance its health-promoting or functional properties through enzymatic techniques. The antioxidant and anti-inflammatory activities observed in RJP within HDFs suggest its potential application in skin health, thus contributing to further augmenting the value of royal jelly protein.

## 5. Conclusions

**Figure 9:**
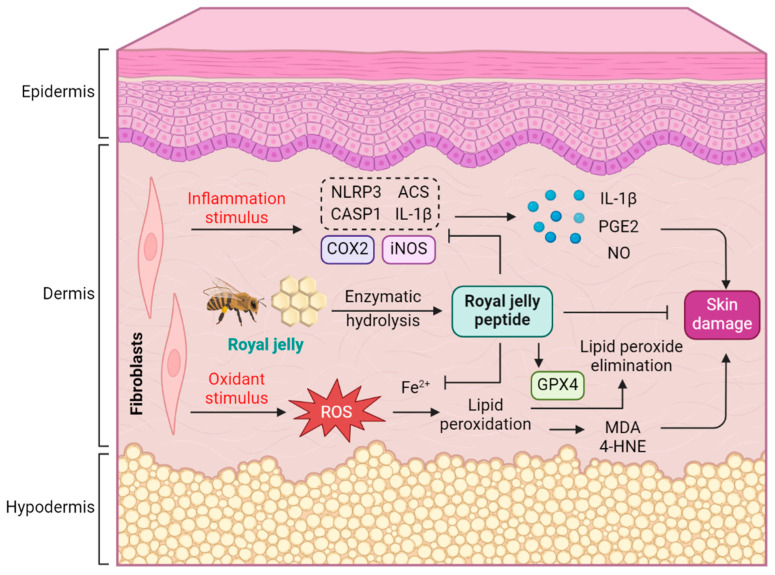
Schematic mechanism of RJP protecting HDFs from oxidative stress and inflammatory damage.

In summary, RJP exhibited a protective effect against H_2_O_2_-induced oxidative stress and AAPH- or *t*-BuOOH-induced lipid peroxidation in HDFs by restoring antioxidative capacity and intracellular iron homeostasis. RJP suppressed NLRP3 inflammasome expression and activation, as well as IL-1β release induced by LPS and ATP, MSU, or nigericin. Additionally, RJP downregulated the expression of COX2 and iNOS in LPS-stimulated HDFs (Figure 9). These findings highlight the antioxidant and anti-inflammatory properties of RJP in HDFs, suggesting its potential application in skin disorders associated with oxidative stress and inflammation, thereby providing novel approaches for enhancing skin health and addressing dermatological concerns.

## Figures and Tables

**Table 1:** Amino acid composition of RJP.

Amino Acid	Composition (%)
Asp	6.97
Thr	1.32
Ser	1.82
Glu	4.21
Gly	1.33
Ala	1.44
Val	2.34
Met	0.40
Ile	3.01
Leu	4.83
Tyr	2.81
Phe	2.20
His	0.59
Lys	1.97
Arg	1.41
Pro	1.75

**Table 2:** Primer sequences in qPCR.

Gene	Species	Sequence (5′-3′)
*NLRP3*	Human	Forward: TGCCCGTCTGGGTGAGA
		Reverse: CCGGTGCTCCTTGATGAGA
*ASC*	Human	Forward: GCCAGGCCTGCACTTTATAGA
		Reverse: GTTTGTGACCCTCGCGATAAG
*CASP-1*	Human	Forward: ATACCAAGAACTGCCCAAGTTTG
		Reverse: GGCAGGCCTGGATGATGA
*IL-1β*	Human	Forward: CCACAGACCTTCCAGGAGAATG
		Reverse: GTGCAGTTCAGTGATCGTACAGG
*COX2*	Human	Forward: CGGTGAAACTCTGGCTAGACAG
		Reverse: GCAAACCGTAGATGCTCAGGGA
*iNOS*	Human	Forward: GCTCTACACCTCCAATGTGACC
		Reverse: CTGCCGAGATTTGAGCCTCATG
*GAPDH*	Human	Forward: GTCTCCTCTGACTTCAACAGCG
		Reverse: ACCACCCTGTTGCTGTAGCCAA

## Data Availability

All data are available on demand.
